# Dendritic Cell-Based Adjuvant Vaccination Targeting Wilms’ Tumor 1 in Patients with Advanced Colorectal Cancer

**DOI:** 10.3390/vaccines3041004

**Published:** 2015-12-11

**Authors:** Shigetaka Shimodaira, Kenji Sano, Koichi Hirabayashi, Terutsugu Koya, Yumiko Higuchi, Yumiko Mizuno, Naoko Yamaoka, Miki Yuzawa, Takashi Kobayashi, Kenichi Ito, Tomonobu Koizumi

**Affiliations:** 1 Center for Advanced Cell Therapy, Shinshu University Hospital, Matsumoto 8621, Japan; E-Mails: kohira@shinshu-u.ac.jp (K.H.); koya@shinshu-u.ac.jp (T.K.); sasa0922@shinshu-u.ac.jp (Y.H.); purimeron@shinshu-u.ac.jp (Y.M.); naoko4152@shinshu-u.ac.jp (N.Y.); m1k1yz@shinshu-u.ac.jp (M.Y.); 2 Department of Laboratory Medicine, Shinshu University Hospital, Matsumoto 8621, Japan; E-Mail: kenjisa@shinshu-u.ac.jp; 3 Shinshu Cancer Center, Shinshu University Hospital, Matsumoto 8621, Japan; E-Mails: takob@shinshu-u.ac.jp (T.K.); tomonobu@shinshu-u.ac.jp (T.K); 4 Department of Breast and Endocrine Surgery, Shinshu University Hospital, Matsumoto 8621, Japan; E-Mail: kenito@shinshu-u.ac.jp

**Keywords:** dendritic cell vaccines, colorectal cancer, Wilms’ tumor 1, immunohistochemistry, tetramer analysis, enzyme-linked immunosorbent spot assay

## Abstract

Despite significant recent advances in the development of immune checkpoint inhibitors, the treatment of advanced colorectal cancer involving metastasis to distant organs remains challenging. We conducted a phase I study to investigate the safety and immunogenicity of Wilms’ tumor (WT1) class I/II peptides-pulsed dendritic cell DC vaccination for patients with advanced colorectal cancer. Standard treatment comprising surgical resection and chemotherapy was followed by one course of seven biweekly administrations of 1–2 × 10^7^ DCs with 1–2 KE of OK-432 (streptococcal preparation) in three patients. Clinical efficacy was confirmed based on WT1 expression using immunohistochemistry on paraffin-embedded tissues and immune monitoring using tetramer analysis and enzyme-linked immunosorbent spot (ELISPOT) assays. WT1 expression with human leukocyte antigen (HLA)-class I molecules was detected in surgical resected tissues. Adverse reactions to DC vaccinations were tolerable under an adjuvant setting. WT1-specific cytotoxic T cells were detected by both modified WT1-peptide/HLA-A*24:02 tetramer analysis and/or interferon-γ-producing cells through the use of ELISPOT assays after the first DC vaccination. Immunity acquired from DC vaccination persisted for two years with prolonged disease-free and overall survival. The present study indicated that DC vaccination targeting WT1 demonstrated the safety and immunogenicity as an adjuvant therapy in patients with resectable advanced colorectal cancer.

## 1. Introduction

Colorectal cancer is the third commonest cancer in males and females and the third leading cause of male and female deaths due to cancer in the United States in 2010, with overall five-year survival rates of 65% for all disease stages and 11% for distant metastasis between 1999 and 2005 [1]. In Japan, colorectal cancer was the third leading cause of cancer death, the first in females and third in males, in 2013. Further, colorectal cancer was the second commonest (fourth in males and second in females) in 2011, with a gradually increasing incidence. The five-year survival rate of colorectal cancer was reported as 70.3% and 67.9% in males and females diagnosed between 2003 and 2005, respectively [2,3].

Despite significant advances in cancer therapy comprising surgical techniques, radiotherapy, and chemotherapy including immune checkpoint inhibitors [4,5,6,7,8,9], the treatment of advanced colorectal cancers with distant metastases involving distant organs remains a substantial clinical challenge.

Antigen-presenting cell-based immunotherapy with active dendritic cells (DCs) has been reported for the induction of efficient immunity against cancer antigens [10]. Cancer vaccines containing autologous monocyte-derived mature DCs conventionally manufactured using granulocyte-macrophage colony-stimulating factor (GM-CSF) and interleukin (IL)-4 are principally targeted against a specific antigen. Wilms’ tumor 1 (WT1) represents the most potent tumor-associated antigen widely detected in cancer, sarcoma, and leukemia in terms of therapeutic function, immunogenicity, specificity, and oncogenicity [11]. DC vaccinations for colorectal cancer have been reported in a proportion of patients [12,13,14,15,16,17]; however, safety and efficacy of DC vaccination therapy primed with WT1 peptides remains unclear.

Human leukocyte antigen (HLA)-restricted WT1 peptides have been shown to be compatible with HLA-A*02:01 and HLA-A*02:06-restricted (126–134: RMFPNAPYL) peptides and peptides restricted to HLA-A*24:02 through modification of the WT1_235–243_ peptide (CYTWNQMNL) by substitution of a second amino acid (methionine: M) with tyrosine (Y). The modified WT1_235–243_ peptide compatible with HLA-A*24:02 can induce cytotoxic T cells (CTLs) to be more effective than the wild-type peptide [18,19,20]. An *ex vivo* technique is being developed to increase the induction of T cells against tumor antigens by DC vaccination. DC vaccines primed with HLA class I-restricted WT1 peptides (WT1-DC) have been shown to be safe and feasible with few adverse reactions in patients with advanced cancers including lung, breast, stomach, biliary tract, pancreas, ovary, and high-grade glioma [21,22,23,24,25,26,27,28]. Phase I clinical trials of DC vaccinations containing the WT1 class II peptide compatible with HLA-DRB1*04:05 (332–347: KRYFKLSHLQMHSRKH) have also been conducted in patients with pancreatic cancer [29]. The efficacy of DC-based immunotherapy is not always demonstrated with conventional evaluation approaches such as the use of the response evaluation criteria in solid tumors (RECIST) [30]. Rather, the clinical efficacy is more evidently demonstrated by the delayed separation of the survival curve with a benefit in terms of prolonged overall survival (OS) [31,32]. The efficacy of DC vaccination may be enhanced by off-target effects of chemotherapeutic drugs, radiotherapy, and chemoradiotherapy [33,34,35]. The combination of chemotherapy and/or radiotherapy has been investigated in addition to the period required for adaptation. Expression levels of cancer-associated antigens with HLA class I and II antigens in tumor tissues may also provide evidence of active immunotherapy against cancers.

Here, we investigated the safety and immunogenicity of DC vaccination targeting WT1 for patients with stage IV colorectal cancer as an adjuvant therapy following surgical resection and chemotherapy.

## 2. Materials and Methods

### 2.1. Manufacture of a DC Vaccine and Vaccination Technique

Mature DCs (mDCs) were generated under Good Gene, Cell and Tissue Manufacturing Practice conditions according to “The Act on the Safety of Regenerative Medicine” introduced in Japan on 25 November 2014 [36]. Immature DCs were generated by culturing adherent cells in AIM-V medium (Gibco, Gaithersburg, MD, USA) containing GM-CSF (50 ng/mL; Gentaur, Brussels, Belgium) and IL-4 (50 ng/mL; R & D Systems Inc., Minneapolis, MN, USA) for 5 days using mononuclear cell-rich fractions isolated through apheresis as previously described [37]. mDCs were differentiated from immature DCs by stimulation with OK-432 (10 μg/mL of streptococcal preparation; Chugai Pharmaceutical Co., Ltd., Tokyo, Japan) and PGE2 (50 ng/mL; Daiichi Fine Chemical Co., Ltd., Toyama, Japan) for 24 h. mDC products were cryopreserved at −152 °C or in the gas layer of a liquid nitrogen tank until the day of administration.

For each vaccination, an aliquot of frozen mDCs was thawed immediately prior to clinical use and primed with 100 μg/mL of good manufacturing practice-grade WT1 peptide (NeoMPS Inc. San Diego, CA, USA) at 4 °C for 30 min, washed twice with removing free peptides, then re-suspended in 1 mL of 1–2 KE of OK-432. WT1 peptides contained HLA A*02:01- or A*02:06-restricted peptides (126–134: RMFPNAPYL), HLA-A*24:02-restricted modified WT1 peptides (CYTWNQML, residue 235–243), and/or class II peptide (332–347: KRYFKLSHLQMHSRKH) compatible with either DRB1*04:05, DRB1*08:03, DRB1*15:01, DRB1*15:02, DPB1*05:01, or DPB1*09:01 [25,29]. One course of seven biweekly sessions was performed with 1–3 × 10^7^ DCs with 1–2 KE of OK-432 intradermally injected at bilateral axillar and inguinal areas per session in accordance with previously described protocols for the clinical use of Gene, Cellular and Tissue-Based Products Manufacturing Products [35,36,37].

### 2.2. DC Vaccine Release Criteria

The antigenic profiles of mDCs were determined using flow cytometry. mDCs were defined as CD11c^+^, CD14^−^, HLA-DR^+^, HL-AABC^+^,CD80^+^, CD83^+^, CD86^+^, CD40^+^, and CCR7^+^ cells [37]. The criteria for DC vaccine administration were as follows: purity defined as >90% proportion of CD11c^+^ CD14^−^ CD86^+^ HLA-DR^+^ >90% cells, >80% viability, mature DC phenotype, negative for bacterial and fungal infection after 14 days, presence of endotoxin ≤0.05 EU/mL, and negative for mycoplasma [37].

### 2.3. Application and Conditions for DC Vaccine Therapy Approved Under “Advanced Medical Care”

(i) Adjuvant therapy after surgical resection or high risk of disease relapse.
(ii) *De novo* cancer at an advanced stage or recurrent cancer after standard therapies.

#### 2.3.1. Competent Standard for DC Therapy and Eligibility

The competent standard for DC therapy and eligibility were as follows: (1) Age, 20–70 years old; (2) performance status, 0/1; (3) no abnormality in organ function, no infectious disease, no blood abnormality, and no bleeding tendency; (4) no history of either cardiovascular disease nor respiratory disorders tolerable for blood apheresis; (5) tolerable to chemotherapy and radiotherapy as standard cancer treatments; and (6) within a period of 6 months after the diagnosis or recurrence of cancer sensitive to chemotherapy.

#### 2.3.2. Evaluation of Safety and Efficacy

Safety evaluations included (1) any allergic reaction after the intradermal injection of the DC vaccine (presence of reduced blood pressure, tachycardia, breathing difficulties, or rash); and (2) local reactions, fever onset, nausea, vomiting, diarrhea, loss of appetite, ulcer of the mucosa, central nervous system damage, anemia, reduced white blood cell count, reduced platelet count, abnormal kidney function, or abnormal liver function during or after the completion of treatment.

We assessed lesions during the course of the treatment using various imaging techniques, including computed tomography (CT), magnetic resonance imaging, and PET, approximately 4 weeks after the completion of DC vaccination. The DC vaccination study was conducted at Shinshu University Hospital and was approved by the Ethics Committee of Shinshu University School of Medicine (approval number 1199, 2 December 2008; 2704, 8 April 2014).

### 2.4. Case Report

#### 2.4.1. Case 1

A 36-year-old woman developed transverse colon cancer with abdominal and mediastinal lymph node metastasis classified as clinical stage IV disease in February 2012. She underwent a right hemicolectomy in March 2012. Pathological findings revealed a T3N4M0 stage IV, EGFR-expressing tumor with wild-type KRAS expression with involvement of the para-aortic, left axillary, and mediastinal lymph nodes. Ten cycles of chemotherapy with folinic acid/fluorouracil and oxaliplatin + bevacizumab (FOLFOX + Bev) were administered between May and October 2012. A complete response according to conventional RECIST was achieved in November 2012. She met the eligibility criteria for DC vaccination therapy as HLA-DNA typing demonstrated a HLA-A*24:02 type compatible with WT1-235 (class I) peptide. DC vaccination containing WT1-235 peptides (a total of 7.64 × 10^7^ DCs; mean, 1.09 × 10^7^ DCs per session) was administered in one course (seven sessions, biweekly) together with oral low-dose chemotherapeutic agents as a metronomic adjuvant therapy (daily 50 mg/day of cyclophosphamide and 2.5 mg of methotrexate, twice weekly) from May to August 2013. No grade 3 or higher National Cancer Institute Common Toxicity Criteria toxicities were associated with vaccination or low-dose chemotherapy. Skin reactions defined as erythema ≥3 cm with induration at intradermal injection sites after 48 h were measured. Grade 1 fever within 48 h of DC vaccination was tolerable. No recurrence of disease or new lesions of colon cancer were noted on CT without the need for additional chemotherapy. Disease-free survival (DFS) evaluated after adjuvant chemotherapy and OS times since the diagnosis of cancer was 34.0 and 43.6 months, respectively.

#### 2.4.2. Case 2

A 41-year-old man presented with occult blood in stool in November 2012. Colonoscopy detected a rectal tumor (Rs, type 2, tub1), and the patient subsequently underwent rectal resection with Group D lymph node resection under laparoscopic examination. White nodular lesions were found on the superficial aspect of the S3 and S4 lever segments using laparoscopy. Lesions identified as liver metastases were resected and pathologically diagnosed as stage IV. During induction of systemic chemotherapy following surgery with bevacizumab in combination with capecitabine and oxaliplatin (CapOx (XELOX) + Bev), he was admitted for DC vaccination. The HLA genotype was confirmed as HLA-A*02:01 and A*24:02 compatible with WT1-126 and modified −235 peptides, respectively. One course (seven sessions, biweekly) of DC vaccination containing WT1-class I peptides (a total of 14.31 × 10^7^ DCs; mean, 2.04 × 10^7^ DCs per session) was administered from July to September 2013 with concurrent chemotherapy. Grade 1 skin reactions of erythema ≥3 cm and induration at the injection sites and grade 1 fever within 48 h of DC vaccination were tolerable, even during chemotherapy, except for persistent grade 2 paresthesia of the legs due to chemotherapeutic drugs. After surgical resection of the rectum and liver followed by DC vaccination in combination with XELOX + Bev, CT imaging indicated no recurrence and complete remission with maintenance chemotherapy with tegafur-gimeracil-oteracil combination (S-1) 1 year after DC vaccination. DFS after surgical resection and OS since the time of diagnosis were 24.5 and 32.4 months, respectively.

#### 2.4.3. Case 3

A 39-year-old woman noticed gradual progression of abdominal fullness as diagnosed with left ovarian metastasis due to rectal cancer 50 months after surgical resection of rectal cancer, the lesion, weighing over 1.3 kg, was resected together with bilateral ovary and uterus in August 2013. She had previously undergone lower anterior resection of the rectum with involvement of peritoneum and lymph nodes as pathological findings of mucinous adenocarcinoma, T4aN2bM1a, stage IV in July 2009, thereafter six cycles of chemotherapy with FOLFOX6 followed by one year-administration of S-1 had been performed. A complete remission has been kept after surgical resection of ovarian metastasis since in November 2013. She met the eligibility criteria for the DC vaccination therapy as HLA-DNA typing demonstrated a HLA-A*24:02 type compatible with WT1-235 (class I) and HLA-DPB1*05:01 type with WT1-332 (class II) peptides. DC vaccination containing WT1-235 and 332 peptides (a total of 7.34 × 10^7^ DCs; mean, 1.05 × 10^7^ DCs per session) was administered in one course (seven sessions, biweekly) together with oral low-dose chemotherapeutic agents (daily 50 mg/day of cyclophosphamide) from February to May 2014. No grade 3 or higher National Cancer Institute Common Toxicity Criteria toxicities were associated with vaccination or low-dose chemotherapy. Skin reactions defined as erythema ≥3 cm with induration at intradermal injection sites after 48 h were measured. Grade 1 fever within 48 h of DC vaccination was tolerable. No recurrences of disease or new lesions were noted on CT without the need for additional chemotherapy. DFS evaluated as secondary remission after resection of metastatic ovarian tumor and OS times since the diagnosis of the rectal cancer was 22.2 and 76.9 months, respectively.

### 2.5. WT1 Expression Using Immunohistochemistry

WT1 expression was evaluated by immunohistochemistry with a mouse monoclonal antibody (6F-H2, DakoCytomation, CA) on paraffin-embedded tumor tissues obtained from the surgical resection of primary cancer tissues as previously described [38]. In parallel with WT1 immunohistochemistry, HLA-ABC antigen (class I, W6/32, DakoCytomation), HLA-DR antigen, alpha-chain (class II, TAL.1B5, DakoCytomation), and epithelial membrane antigen (EMA; Clone E29, DakoCytomation) immunohistochemistry were also performed on the same cancer tissues.

### 2.6. Immune Monitoring with Tetramer Analysis and Enzyme-Linked Immunosorbent Spot (ELISPOT) Assays

Freshly isolated peripheral blood mononuclear cells (PBMCs) were stained with PE-conjugated human immunodeficiency virus (HIV)/HLA-A*24:02 tetramers as a negative control or PE-conjugated WT1-modified peptides/HLA-A*24:02 tetramers (MBL, Medical & Biological Laboratories Co., Ltd., Nagoya, Japan). Other stains included allophycocyanin-conjugated anti-CD3 mAb and fluorescein isothiocyanate-conjugated anti-CD8 mAb prior to analysis by flow cytometry (BD FACSCalibur™). The presence of WT1 antigen-specific CTLs (WT1-CTLs) was defined as previously described [39].

ELISPOT assays were performed to measure WT1-specific interferon (IFN)-γ production by PBMCs using precoated human IFN-γ ELISPOT PLUS kits (Mabtech, Nacka Strand, Sweden). Isolated PBMCs, including effector and stimulator cells, were isolated and cultured (1 × 10^6^ cells/well) in the presence of either WT1_126–134_,WT1_235–243_ or WT1_332–347_ peptide for 16–20 h. Experiments were performed in duplicate. The presence of WT1-specific CTLs was confirmed by counting with an automated ELISPOT reader (Autoimmun Diagnostika, Strassberg, Germany) according to the following criteria: (1) at least 15 WT1-specific spots per 1 × 10^6^ PBMCs; and (2) at least 50% more WT1-specific spots than negative peptide (HIV peptide) spots [39]. The Dunnett's test was used for multiple comparisons after the course of DC vaccination as contrast in before vaccination on duplicated ELISPOT assays. *p* < 0.05 indicates statistically significance and was calculated using IBM SPSS Advanced Statistics ver 23.0 (IMB Japan, Tokyo, Japan).

## 3. Results

### 3.1. WT1 Expression Using Immunohistochemistry

**Figure 1:**
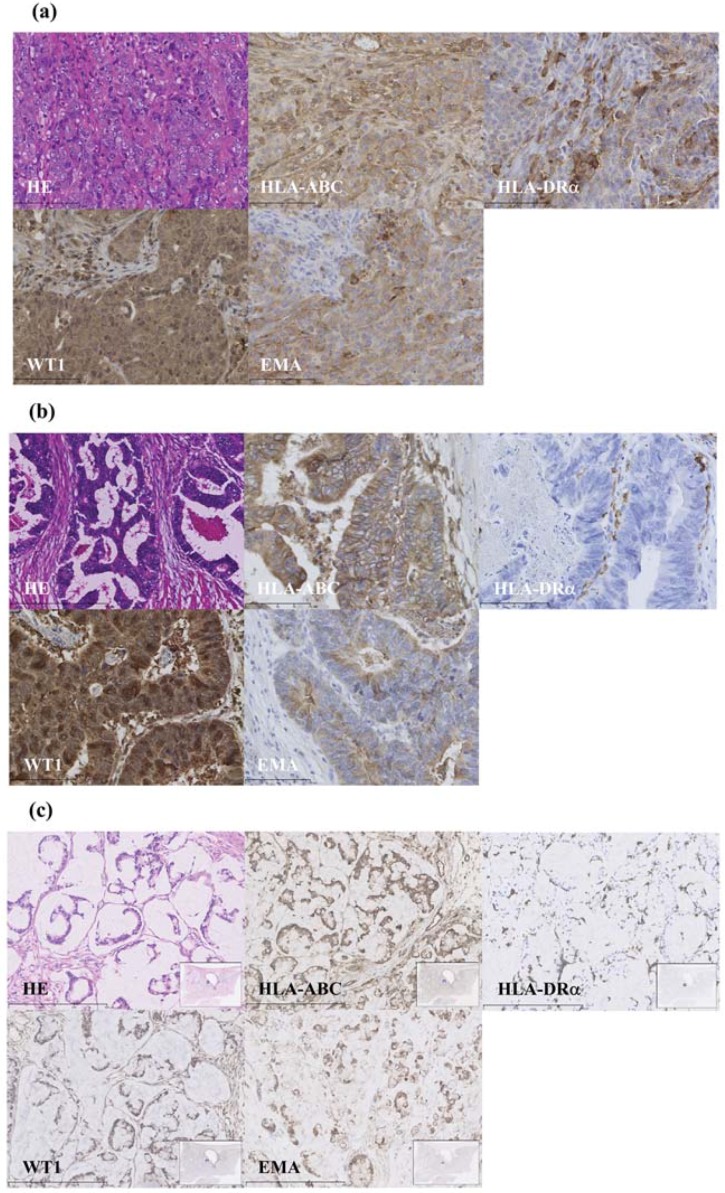
Primary cancer immunohistochemistry: (**a**) Poorly differentiated adenocarcinoma of the colon from case 1 expressing WT1^++^ HLA-class I^+^ HLA-class II^+^ EMA^+^; (**b**) well-differentiated adenocarcinoma of the rectum from case 2 expressing WT1^++^HLA-class I^+^HLA-class II^−^EMA^+^; and (**c**) mucinous adenocarcinoma of the rectum from case 3 expressing WT1^+^HLA-class I^+^HLA-class II^+^EMA^+^. WT1, Wilms’ tumor 1; HLA, human leukocyte antigen; EMA, epithelial membrane antigen.

Strong nuclear and cytoplasmic expression of WT1 was observed in the primary cancer cells, as shown in Figure 1a–c for cases 1, 2, and 3, respectively. EMA and HLA-class I staining of the same specimens were moderate to weakly positive in either case. HLA-class II was positive in case 1 and dimly positive in case 3, but not in case 2. These findings indicate WT1-CTLs primed with DC vaccinations targeting WT1 were highly sensitive to colorectal cancer cells. It also suggested potential reaction to rectal cancer cells through helper T cells in case 3, which were applied together with the WT1-class II helper peptide.

### 3.2. Immune Monitoring with Tetramer Analysis and ELISPOT Assays

**Figure 2:**
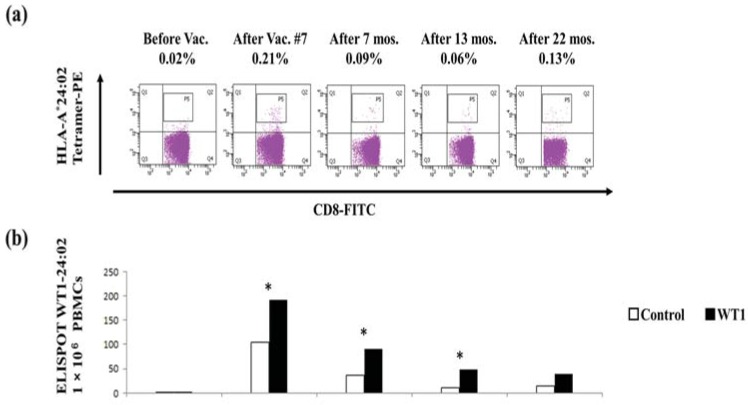

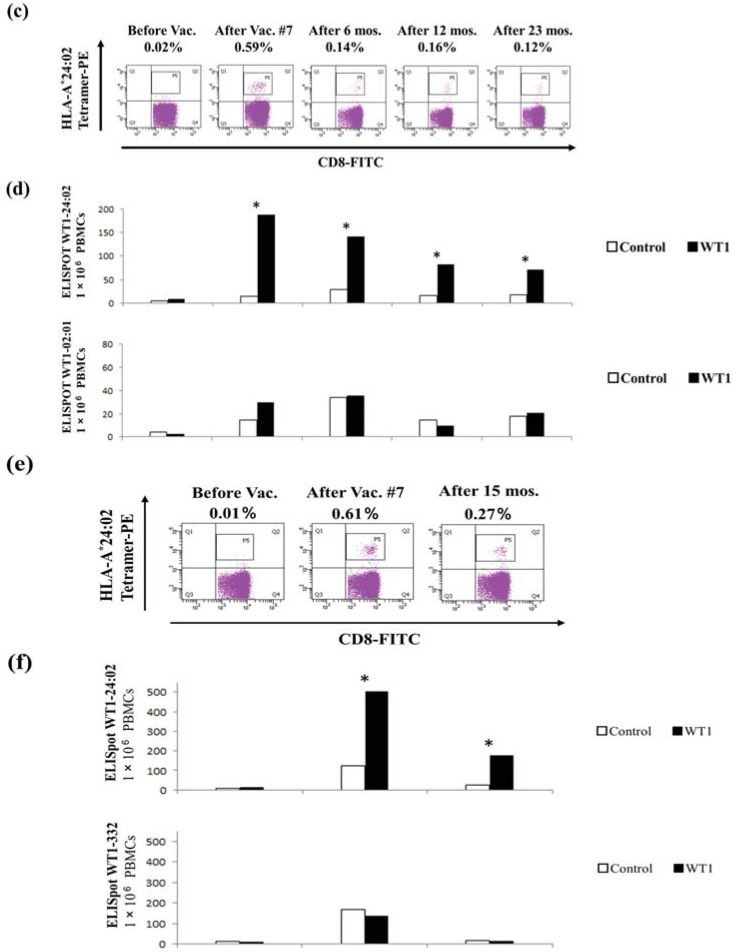
WT1-CTLs induced by WT1_126–134_ (HLA-A*02:01), WT1_235–243_ (HLA-A*24:02), or WT1_332-347_ peptide-pulsed DC vaccination: (**a**) modified WT1-peptide/HLA-A*24:02 tetramer analysis from case 1. Percentages represent the proportion of tetramer-positive cells in the total CD8^+^ T-cell population; (**b**) interferon (IFN)-γ-producing clones on ELISPOT assays with WT1_235–243_ peptide (HLA-A*24:02) from case 1; (**c**) comparison of WT1-peptide/HLA-A*24:02 tetramer analyses before and after DC vaccination from case 2; (**d**) IFN-γ-producing clones on ELISPOT assay with WT1_235–243_ peptide (HLA-A*24:02; upper panel) and WT1_126–134_ peptide (HLA-A*02:01; lower panel) from case 2; (**e**) modified WT1-peptide/HLA-A*24:02 tetramer analysis from case 3; and (**f**) IFN-γ-producing clones on ELISPOT assay with WT1_235–243_ peptide (HLA-A*24:02; upper panel) and WT1_332–347_-class II peptide (HLA-DPB1*05:01; lower panel) from case 3. WT1, Wilms’ tumor 1; CTL, cytotoxic T cell; HLA, human leukocyte antigen; DC, dendritic cell; IFN, interferon; ELISPOT, enzyme-linked immunosorbent spot; * *p* < 0.05 indicates statistical significance using Dunnett’s test; Black bars, WT1 and white bars, negative control.

In case 1, WT1-CTLs were determined by both modified WT1-235 peptide/HLA-A*24:02 tetramer analysis and the presence of IFN-γ-producing clones on ELISPOT assays during one course of DC vaccination and at approximately six-month intervals up to two years after one course without maintenance chemotherapy. After one course of DC vaccination, the immune monitoring assay demonstrated WT1-CTLs comprised 0.21% of the CD8^+^ T-cell population (Figure 2a) with demonstration of a specific number of IFN-γ-spots using ELISPOT assays (Figure 2b) according to the positive criteria [39]. WT1-tetramer analysis and ELISPOT assays demonstrated a specific number WT-CTLs persisted up to 22 months after DC vaccination. However, the latter assay demonstrated a gradual decrease in the number of IFN-γ-specific spots, in contrast to the persistent low ratio of WT1-CTLs to CD8^+^ T cells.

WT1-CTLs were determined by modified WT1-235 peptide/HLA-A*24:02 tetramer analysis after DC vaccination and found to represent 0.59% of all CD8^+^ T cells in case 2 (Figure 2c). Although the proportion of WT1-CTLs gradually decreased, WT1-CTLs were found to be present two years after DC vaccination. IFN-γ-producing clones on ELISPOT assays after one course of DC vaccination demonstrated WT1-specific spots stimulated with both WT1-126 compatible with HLA-A*02:01 and WT1-235 peptides (Figure 2d), in accordance with previously described criteria for positivity [39]. The former panels indicated a loss of specificity, with no difference observed between negative controls and test samples after one DC vaccination course, and the latter panels demonstrated a gradual decrease in the number of WT1-positive spots at two years.

In case 3, WT1-CTLs were determined by modified WT1-235 peptide/HLA-A*24:02 tetramer analysis after DC vaccination and found to represent 0.61% of all CD8^+^ T cells (Figure 2e), concomitant with ELISPOT assays with WT1-235 peptide (Figure 2f, upper panel). The proportion of WT1-CTLs gradually decreased to be present at 0.27% 15 months after DC vaccination as is the same in other two patients. IFN-γ-ELISPOT assays using WT1-332 (HLA-Class II peptide) after one course of DC vaccination demonstrated over 100 numbers with low specificity of spots (Figure 2f, lower panel).

## 4. Discussion

Colorectal cancer is the third leading cause of cancer death in the United States and Japan. Further, colorectal cancer with distant metastasis still confers a poor prognosis as demonstrated by the significantly reduced five-year OS rate [1]. In the present study, cases 1 and 2 with stage IV disease at the time of diagnosis have a DFS of 34.0 and 24.5 months, respectively, and an OS of 43.6 and 32.4 months, respectively. Despite a long observation period, both cases exhibited long relapse-free survival following DC vaccination.

HLA molecules harbor cancer antigens that promote the binding of DCs to receptors on CD8^+^ killer and CD4^+^ helper T cells leading to an immune response against cancer cells. Sources of antigens for clinical vaccination include peptides, lysates, tumor cells, and mRNAs [40]. DC vaccinations for colorectal cancer primed with carcinoembryonic antigen (CEA) peptide and mRNA have been reported to induce CEA-specific CTLs exhibiting disease stability and response in a proportion of patients [12,13,14,15,16,17]. WT1 and HLA antigens may also provide a common target of DC vaccination therapy for heterogeneous colorectal cancer. Despite varying expression levels of WT1 antigen, 69% of cases with colorectal cancer could be detected using the same monoclonal antibody (6F-H2), with a particularly high ratio observed in well-to-moderately differentiated colorectal adenocarcinoma (76%) compared with poorly differentiated tumors (38%) [38]. In the present study, WT1 antigens were found to be strongly expressed in colorectal adenocarcinoma tissues in addition to HLA-ABC antigens in both cases (Figure 1), likely representing an advantageous on-target effect of DC vaccination.

The efficacy of DC vaccination is likely attributable to the inhibition of immune suppressors other than regulatory T cells, tolerogenic DCs, and myeloid-derived suppressor cells, which are derived from autoreactive and cancer-associated mechanisms [41,42,43,44,45,46]. The efficacy of chemotherapeutic drugs with off-target effects and radiotherapy may be due to ablation of immune suppressive mechanisms in the tumoral microenvironment [33,34], which would consequently accelerate the induction of WT1-CTLs. Chemoradiotherapy has also been shown to enhance the induction of WT1-CTLs in patients receiving WT1-targeted DC vaccinations for pancreatic cancer [35]. The identification of the most appropriate therapies combining DC vaccination with radiotherapy and chemotherapy with off-target effects, in addition to the blockade of immune checkpoints [47], would be expected to improve the outcomes of advanced stage colorectal cancer. Our preliminary study of patients with colorectal cancer after the standard therapies has several limitations such as the small sample size and a group of heterogeneous patients. However, the DC vaccination targeting WT1 as an adjuvant setting in the course of standard therapies may be feasible and well tolerable for advanced colorectal cancer. Evaluation of the clinical course suggested that DC vaccination has efficacy and beneficial effects for some patients. The efficacy and safety of DC vaccination should be determined using large number of a phase II prospective trials for colorectal cancer.

Immune monitoring of DC vaccination, using validated tetramer analysis and ELISPOT assays, is important as a proof of concept in clinical studies and trials. As high expression of wild-type WT1 has been reported in cancer cells with oncogenesis [48], the modified WT1 peptide for HLA-A*24:02 has been proven to be more effective than HLA-A*02:01 [18,19,20]. It is an interesting phenomenon that WT1-CTLs induced with the modified WT1 (WT1_235–243_) peptide persist for up to two years after DC vaccination (Figure 2a–f), with a different trend observed compared with the use of the wild-type WT1 peptide (WT1_126–134_ or WT1_332–347_) in the same patient (Figure 2d,f). The activity of WT1-CTLs induced by the modified WT1 peptide commonly decreased, as shown by the results of the ELISPOT assays (Figure 2b,d,f).

Further studies are required to evaluate whether effecter memory T cells naturally decrease in number in response to the disease-free state and whether PD1-positive (exhausted CTLs) are influenced by DC vaccination [25]. Future studies evaluating DC vaccines pulsed with WT1-class I/II peptides may demonstrate the induction of WT1-CTLs primed against WT1 and HLA-class I/II-positive colorectal cancers. Prospective clinical trials are required to evaluate the efficacy of acquired immunity in response to adjuvant DC vaccination in improving the prognosis of advanced colorectal cancer.

## 5. Conclusions

A phase I study of WT1 peptides-pulsed DC vaccination for patients with advanced colorectal cancer demonstrated the safety and immunogenicity as an adjuvant setting. Based on the results of our pilot study, we should continue the designated phase II clinical trials in patients with colorectal cancer.
